# Current implications of cyclophilins in human cancers

**DOI:** 10.1186/1756-9966-29-97

**Published:** 2010-07-19

**Authors:** Jinhwa Lee, Sung Soo Kim

**Affiliations:** 1 Department of Biomedical Laboratory Science, Dongseo University, Busan 617-716, Korea; 2 Department of Biochemistry and Molecular Biology, Medical Science and Engineering Research Center for Bioreaction to Reactive Oxygen Species (BK-21) and Biomedical Science Institute, School of Medicine, Kyung Hee University, Seoul 130-701, Korea

## Abstract

Cyclophilins (Cyps), the intracellular receptor for immunosuppressant cyclosporine A (CsA), play important cellular roles through activities of peptidyl-prolyl cis-trans isomerase (PPIase) and chaperones. Cyps are structurally conserved and found in both prokaryotic and eukaryotic organisms, including humans which contain 16 Cyp isoforms. Although human Cyps were identified about 25 years ago, their physiological and pathological roles have only been the focus of attention recently because of their possible involvement in diseases and ailments such as HIV infection, hepatitis B and C viral infection, atherosclerosis, ER stress-related diseases and neurodegenerative diseases, *etc*. There are reports for upregulated Cyps in many human cancers and there are also strong correlations found between Cyps overexpression and malignant transformation. This review discusses the important and diverse roles of Cyps overexpression in human cancers. Understanding biological functions of Cyps will eventually lead to improved strategies for cancer treatment and prevention.

## Introduction

Cyclophilins (Cyps) were initially identified as biological receptors for the immunosuppressive drug cyclosporine A (CsA) approximately 25 years ago. Later, they were shown to have peptidyl-prolyl cis-trans isomerase (PPIase) enzymatic activity which catalyzes cis-trans isomerization of peptide bonds preceding proline [1-6]. Cyps also possess chaperone activities. These two functions allow Cyps to be involved in proper folding of proteins in combination with other proteins. Although CsA is an effective inhibitor of Cyps, immunosuppressive activity of CsA is not the result of inhibition of the Cyps' activities. Rather, the Cyp-CsA complex accidentally inhibits calcineurin activity and thereby suppresses T-cell proliferation by interfering with downstream signal transduction [7].

Cyps are highly conserved from *E. coli *to humans throughout evolution. A total of 16 Cyp isoforms have been found in humans [8], but 7 major human Cyp isoforms, namely hCypA, hCypB, hCypC, hCypD, hCypE, hCyp40, and hCypNK [9], have been well characterized. They play diverse roles by localizing through unique domains for particular cellular compartments including the cytosol, endoplasmic reticulum (ER), mitochondria and nucleus. The clinical importance of Cyps has been implicated in diverse pathological conditions including HIV [10], hepatitis B and C viral infection, atherosclerosis [11,12], ER stress-related diseases such as diabetes, and neurodegenerative diseases. Cyps are also involved in normal cellular functions of muscle differentiation, detoxification of reactive oxygen species (ROS) [13], and immune response [14]. Their novel and unfamiliar nuclease activity similar to apoptotic endonucleases suggests a potential role in apoptotic DNA degradation. Overall roles of Cyps may encompass far more than already defined functions such as protein folding.

CypA overexpression in diverse types of cancer has been recently reported by many research groups. Subsequently, overexpression of other Cyps has also been repeatedly observed in various cancers. Although Cyps expression levels and patterns in many cancer types have been considerably well documented, the precise roles of Cyps in cancer are hardly defined. Here, we will discuss the implications of Cyps in cancer biology and particularly give emphasis on CypA that has been studied most extensively in diverse human cancers. Better understanding of Cyps' function in cancers may divulge their potential applications in cancer prevention, diagnosis, and treatment.

## Regulation of Cyclophilin A gene expression in human cancers

After the initial finding of upregulation of CypA in hepatocellular carcinoma [15,16], CypA has been reported to be overexpressed in small cell lung cancer [17-20], pancreatic cancer [21-25], breast cancer [26,27], colorectal cancer [28-30], squamous cell carcinoma [31,32], melanoma [33], and glioblastoma multiforme [34]. In addition to CypA's automatic malregulation in diverse cancers, CypA can be influenced in its expression by chemotherapeutic agents. Independent research groups demonstrated that treatment with chemotherapeutic agents, 5-aza-2-deoxycytidine (DAC), celecoxib, and 5-fluorouracil (5-FU), lowers CypA expression [[21,29] and [30]]. On the contrary, our group found that cisplatin causes CypA overexpression and induces resistance to diverse chemotherapeutic agents including cisplatin (unpublished data). Upregulation of CypA in cancer is not so unusual; yet the exact mechanisms of transcriptional alteration of CypA in cancer are still elusive.

Initially, CypA gene together with those of glyceraldehyde 3-phosphate dehydrogenase, rRNA and beta-actin was considered one of the constitutively expressed house- keeping genes which do not respond to external stimuli. Considering the chaperone activity of CypA protein, it is not surprising to find up-regulation of CypA gene in response to stresses that can cause protein damage or denaturation [35]. Since molecular regulatory mechanisms of CypA expression are poorly understood, it needs to be further studied whether the CypA up-regulaion in cancer is controlled by the same regulatory mechanisms of stress induction.

If up-regulation of CypA in cancers is linked to p53 and HIF-1α, most well-characterized cancer-related transcriptional regulatory factors, has been sought by several groups. Choi *et al*. demonstrated that HIF-1α can upregulate CypA by HIF-1α binding to hypoxia response elements (HRE) in the CypA promoter region under hypoxic conditions [36]. Similarly, Gu *et al*. first showed that CypA is up-regulated during p53-induced apoptosis using quantitative proteomic profiling [37,38]. They also proposed that transcription of CypA might be induced by activated p53. While no direct evidence has been reported that p53 is activated or stabilized by CypA, it is interesting to note that PIN 1, another type of PPIases, stabilizes p53 through affinity binding of PIN 1 to the p53's proline rich domain (PRD) [39]. Our group recently discovered binding activity of CypA to p53 which leads to stabilization of p53 (unpublished data).

## Clinical implications of the overexpressed Cyclophilin A in cancers

Upregulation of CypA in many cancer types dictates an advantage of CypA overexpression toward cancer development. While the exact roles of CypA in cancer cells are yet to be defined, understanding the precise function of CypA during tumor development will be critical to assess its potential as a target for therapeutic intervention.

Positive growth effect by excessive CypA on cancer cells was first reported by Howard *et al*. They showed that overexpression of CypA in small cell lung cancer stimulates cancer cell growth, and knockdown of CypA slows cancer cell growth, independent of its effects on angiogenesis [17,18]. Other roles of CypA have also been proposed. Qi *et al*. suggested that CypA is upregulated during malignant tansformation of esophageal squamous cells [32]. CypA abundance is more than 5 fold, compared to non-malignant immortalized control cell lines [40]. There also exist reports that CypA may regulate metastasis [32,33].

During development of solid tumors, ROS are continuously generated in tumor's central hypoxic region. Hong *et al*. suggested that CypA has antioxidant effects through its PPIase activity [13]. It is consistent with the finding that CypA overexpression promotes cancer cell proliferation and blocks apoptosis induced by hypoxia [36]. Choi *et al*. showed that overexpression of CypA in cancer cells renders resistance to hypoxia- and cisplatin-induced cell death in a p53 independent manner [36].

There are several reports suggesting that inhibition of PPIase activity of CypA may generate potential chemotherapeutic effects. Yurchenko *et al*. has reported that cell surface expression of CD147, tumor cell-derived collagenase stimulatory factor, is regulated by CypA [41,42]. Overexpressed CypA interacts with the proline-containing peptide in CD147's transmembrane domain and stimulates human pancreatic cancer cell proliferation [43]. Zheng *et al*. also demonstrated in breast cancer cells that prolactin needs to bind CypA for cancer progression and tumor metastasis [44]. Han *et al*. showed that CsA and sanglifehrin A (SfA), two CypA inhibitors, increase chemotherapeutic effect of cisplatin in glioblastoma multiforme [34]. Overexpression and known functional roles of CypA in various cancer types are summarized in Table 1.

**Table 1:** Cyclophilin A in human cancers

Cancer type	Functions and implications of CypA in cancers	Contributers
**Lung cancer**	The first identification of CypA overexpression in lung cancer	**Campa et al., **Cancer Res. (2003)
	Potential role of CypA in early neoplastic transformation and as a biomarker	**Howard et al., **Lung Cancer (2004)
	Regulation of cancer growth, angiogenesisa and apoptosis through CypA knockdown and overexpression	**Howard et al., **Cancer Res. (2005)
	Role of exogenous CypA in increased H446 cell growth through ERK1/2 pathway activation	**Yang et al., **BBRC (2007)

**Pancreatic cancer**	Identification of CypA as a decreased factor by 5-aza-2-deoxycytidine	**Cecconi et al., **Eletrophoresis (2003)
	Involvement of increased CypA in pancreatic carcinogenesis	**Shen et al., **Cancer Res. (2004)
	Effect on the gene expression of several key molecules including NRPs, VEGF, and VEGFRs	**Li et al., **Am J Surg (2005)
	Stimulation of cancer cell proliferation by increased CypA through CD 147 signaling	**Li et al., **Cancer Res (2006)
	Association of increased CypA with tumor invasion, metastasis, and resistance to therapy	**Mikuriya et al., **Int J Oncol (2007)

**Hepatocellular carcinoma**	Regulation of cancer cell proliferation and increase of hepatocarcinoma formation by interaction of increased CypA with calcineurin	**Corton et al., **Cancer Let (1998)
	Identification as a useful HCC marker in tumor tissues	**Lim et al., **BBRC (2002)

**Breast cancer**	Assessment of CypA down-regulation through proteomics in melphalan-resistant and -susceptible MCF-7 cell lines	**Hathout et al., **J proteomic Res (2002)
	Role of CypA in cancer cell progression and regulation of JAK2	**Zheng et al., **Cancer Res (2008)

**Colorectal Cancer**	Identification of association of CypA with tumor development and tumor progression through protein profiling	**Melle et al., **Int J Mol Med (2005)
	Role of CypA in COX-2-independent chemopreventive effect by celecoxib	**Lou et al., **Cancer Epidemiol (2006)
	Upregualtion of CypA among5-fluorouracil (5-FU) response proteins for CRC chemotherapy	**Wong et al., **Oncol Rep (2008)

**Squamous cell carcinoma**	Involvement in oncogenesis in SCC	**Chen et al., **Proteomics (2004)
	Possible role as a malignant transformation-related protein in ESCC	**Qi et al., **J Cell Biochem (2008)

**Melanoma**	High level expression in primary and metastatic melanoma	Al-**Ghoul et al., **J Proteome Res (2008)

**Prostate cancer**	Preventing hypoxia- and cisplatin-induced apoptosis	**Choi et al., **Cancer res (2007)

**Glioblastoma multiforme**	Increasing expression of CypA in human glioblastoma multiforme	**Han et al., **Oncol Rep (2010)

## Other cyclophilins and cancers

Other Cyps including CypB, CypC, CypD and Cyp40 might also play important roles in carcinogenesis. Kim *et al*. reported that CypB protects cells against ER stress-induced cell death at least partly through blocking the Ca^2+ ^leakage from ER to cytosol [45]. Overexpression of CypB is associated with tumor progression through regulation of hormone receptor expression and gene products involved in cell proliferation and motility [46]. Interestingly, CypB possesses two antigenic epitopes (CypB (82-92) and CypB (91-99)) recognized by HLA-A24-restricted and tumor-specific cytotoxic T lymphocytes that are suggested to be used for vaccines against cancers [47].

CypC is another Cyp family member that is primarily located in ER, but its role remains to be determined. CypC can form a complex with the COOH-terminal fragment of osteopontin. This complex binds to CD147 to activate Akt1/2 and MMP-2 in 4T07 murine breast cancer cells. This CyC- osteopontin complex regulates *in vitro *migration and invasion properties of 4T1 and 4T07 breast cancer cells [48].

CypD is an important component of the mitochondrial permeability transition pore, another components of which are the voltage-dependent outer membrane anion channel, adenine nucleotide translocator [49,50], and hexokinase. PPIase activity of CypD may be necessary for binding of CypD to the MPTP complex [51]. Although function of CypD in mitochondria is controversial, overexpression of CypD attenuates sensitivity of HEK 293 and rat glioma C6 cells to apoptotic stimuli, with protective effects of CypD requiring PPIase activity [52]. Consistently, several reports have shown that CypD is overexpressed and has an anti-apoptotic effect in various tumors via a Bcl 2 collaborator and an inhibitor of cytochrome c release from mitochondria [53]. This protective effect is independent of the MPTP [53].

Cyp40 mRNA has also been reported to increase in many breast cancer cell lines including MCF-7 [54]. Additionally, Cyp40 mRNA also increases in response to high temperature stress in MCF-7 cells [55]. Up-regulation of Cyp40 is reported to be correlated with oxidative stress in MCF-7 cells and prostate cancer cell lines. Genetic analysis of breast cancers shows 30% allelic loss of Cyp40 from patients heterozygous for Cyp40 [56]. Overexpression and potential roles for other Cyps in various cancer types are summarized in Table 2.

**Table 2:** Other cyclophilins in human cancers

Cancer type	Isoforms	Implications in cancers	Contributers
**Breast cancer**	CypB	A transcription inducer	**Fang et al., **Am J Pathol. (2009).

**Breast cancer**	Cyp40	Having important functional implications for ER alpha and other steroid receptors in breast cancer	**Eliseev etal., **J Biol Chem. (2009)
		Increasing in response to high temperature stress	**Machida etal., **J Biol Chem. (2006)

**Breast cancer**	CypC	Binding to osteopontin via CD147 and increase in migration and invasion	**Mi Z et al., **Cancer Res. (2007)

**Tumors of the breast, ovary, and uterus**	CypD	Inhibition of PT-pore	**Marzo et al., **Cancer Res. (2007)
		Interacton with Bcl2	**Eliseev etal., **J Biol Chem. (2009)

## Summary

Cyps regulate protein folding through PPIase enzymatic and chaperone activities in specific locales of the cells to ensure correct conformation and to counterbalance conformational variations under diverse stress conditions. In addition to PPIase and chaperone activities, each isoform of Cyps has other specific intracellular and extracellular roles. Although roles of Cyps have recently been explored in more details, many physiological and pathological aspects of Cyps' biology still remain unclear.

CypA among the Cyps was first reported to be upregulated in tumors, including small cell lung cancer, pancreatic cancer, breast cancer, colorectal cancer, squamous cell carcinoma, glioblastoma multiforme, and melanoma. This wide spectrum of cancers harboring excess CypA denotes an important role of CypA in tumor development. The possible roles of CypA in cancers might involve increased cell proliferation, blockage of apoptosis, malignant transformation, angiogenesis, metastasis, and resistance to chemotherapeutic agents. Transcriptional upregulation of CypA mediated by p53 and HIF-1α during tumor development would magnify the cancer-prone effect of CypA.

Some groups have proposed CypA as a cancer biomarker for certain cancer subtypes because expression levels nicely correlate with tumor progression. Although less informed at now, other Cyps are also known to be overexpressed and proposed to be involved in various cancers.

CsA and SfA induce apoptosis in various cancer cells via inhibition of PPIase activity of Cyps, and have been tested for clinical applications in diverse cancer types [34]. However, CsA and Sfa can hardly be applied to cancer patients because of immunosuppressive effects. The detailed understanding on the molecular mechanisms by which Cyps affect cancer development will aid the development of new chemotherapeutic agents. Specific inhibitors of the PPIase activity of Cyps devoid of immune suppressive effects will be promising for the treatment of cancers currently resistant to available chemotherapeutics.

## Competing interests

The authors declare that they have no competing interests.

## Authors' contributions

JL and SSK read and approve the final manuscript.
